# Reduced graphene oxide on silicon-based structure as novel broadband photodetector

**DOI:** 10.1038/s41598-021-92518-z

**Published:** 2021-06-21

**Authors:** Carmela Bonavolontà, Antonio Vettoliere, Giuseppe Falco, Carla Aramo, Ivo Rendina, Berardo Ruggiero, Paolo Silvestrini, Massimo Valentino

**Affiliations:** 1 Istituto Scienze Applicate e Sistemi Intelligenti “E. Caianiello” ISASI-CNR, Comprensorio “A. Olivetti” Ed. 70, 80072 Pozzuoli, Naples, Italy; 2 Istituto Nazionale Fisica Nucleare INFN Sez. Napoli Complesso Universitario, Monte Sant’Angelo Ed 6, 80126 Naples, Italy; 3 Dipartimento di Matematica e Fisica DMF, Università Della Campania “Luigi Vanvitelli”, 81100 Caserta, Italy

**Keywords:** Optical properties and devices, Graphene, Electronic properties and devices, Materials for devices

## Abstract

Heterojunction photodetector based on reduced graphene oxide (rGO) has been realized using a spin coating technique. The electrical and optical characterization of bare GO and thermally reduced GO thin films deposited on glass substrate has been carried out. Ultraviolet–visible–infrared transmittance measurements of the GO and rGO thin films revealed broad absorption range, while the absorbance analysis evaluates rGO band gap of about 2.8 eV. The effect of GO reduction process on the photoresponse capability is reported. The current–voltage characteristics and the responsivity of rGO/n-Si based device have been investigated using laser diode wavelengths from UV up to IR spectral range. An energy band diagram of the heterojunction has been proposed to explain the current versus voltage characteristics. The device demonstrates a photoresponse at a broad spectral range with a maximum responsivity and detectivity of 0.20 A/W and 7 × 10^10^ cmHz/W, respectively. Notably, the obtained results indicate that the rGO based device can be useful for broadband radiation detection compatible with silicon device technology.

## Introduction

The photodetectors are of great interest in several technological applications thanks their capability to convert an optical signal into electrical signal through the light-matter interactions^1^. In particular, broadband photodetectors are used in multiple applications such as environmental monitoring^2^, imaging^3^, fire detection^4^, astronomical observations^5,6^, remote sensing^7,8^ and recently in the military field for missile detection^9^. One of the main limitation for a broadband photodetector could be represented by its poor capability to detect UV radiation.

Wide band-gap semiconductors (WBG) such as (SiC, GaN, ZnO, TiOX^10–14^ are currently considered the best candidates for UV photodetection, due to their chemical stability and high resistance, but their insensitivity to visible light reduce their field of applications.

In addition, silicon is a widely used semiconductor material for photodetectors due to its low bandgap, low surface states, reliability, nature production, and high-speed detection capability^15–17^. However, in the UV region, also silicon photodetectors face a great challenge. It consists of the current low photoresponsivity, typically less than 0.1 A/W for λ < 400 nm, caused by a high reflection coefficient and a low penetration depth of UV light into the material. For example, typically a silicon pn junction has a depth greater than 200 nm^18^. Since the penetration depth of UV light for λ < 370 nm into the silicon is less than 20 nm^19^, the photo-generated carriers are mainly close to the silicon surface and therefore they need a diffusion length in the material on a scale of about 100 nm in the region near the junction. When this condition is not satisfied, a significant recombination of the carriers is determined, responsible for a low photo-generated current, so that a limitation of the sensor performance occurs.

To obtain high-performance broadband silicon-based photodetectors it is necessary to improve the UV radiation detection. To this purpose an ultra-surface junction with efficient separation and charge collection running at high speed, is required. Semi-transparent metal structures, such as Si-Schottky, can only partially meet the requirement, as a large percentage of UV light is reflected or absorbed by the metal layer without contributing to the photocurrent, resulting in low photosensitivity^20^.

Recently, graphene (Gr) characterized by a single layer of carbon, has shown a broad absorption spectrum covering the ultraviolet to the far-infrared^21,22^. Moreover it shows excellent electronic conductivity^23^ and optical transmittance^24^, its electronic properties show great potential to replace metals as transparent electrodes^25,26^. Then, graphene has a maximum thermal conductivity of 20 W/cm K, ten times greater than that of silicon 1.5 W/cm K, and thus can effectively facilitate rapid heat dissipation and improves temperature stability mainly when working in UV radiation exposure mode for a long time.

Therefore, Gr has been drawn attention to develop graphene-based broadband photoconductors, so that structures formed by Gr/Si in solar cells have been proposed^27–29^, showing excellent performance. The ultra-surface Gr/Si Schottky structure may be more suitable for UV detection, because it not only solves the problem of high carrier recombination, due to the surface junction satisfying the light UV penetration depth requirements in the silicon, but it also simplifies the manufacturing process reducing the production cost of devices.

In addition, two important derivatives of graphene such as graphene oxides (GOs) and reduced graphene oxides (rGOs) have been taken into account to realize broadband photodetectors thanks their broad-spectrum light absorption^30^, and their excellent physical and chemical properties, that might find important applications in electronics and optics.

We propose here the graphene oxide (GO) as a novel candidate to replace the graphene in the realization of a broadband photodetector, because of its excellent electronic conductivity and optical transmittance. Basically, the graphene oxide (GO) is a substance known for almost 150 years, produced through the oxidation of graphene/graphite by attaching oxygen-containing functional groups to graphene. It has attracted great interest due to its availability and production over a wide area, solution-based processing and attractive semiconductor properties ^31^. Meanwhile, existence of various oxygen-containing functional groups enables GO as a promising prospect in energy and environmental science, as well as biotechnology^32^.

Therefore, functionally reduced GO could be produced through the reduction of GOs to tailor the surface functional groups and to be compatible with several substrates. In addition, the rGO solubility ensures that flexible thin films with large area could be realized^33^. Moreover, the possibility to tune the electrical and optical properties of GO, by means of chemical and thermal reduction treatments, has provided the GO-based thin films as good candidates in the realization of photovoltaic and nonvolatile memory devices, thin-film transistors (TFTs) and transparent conductors^34,35^.

For this reason, great efforts have been made to optimize the manufacturing process to improve the so-called exfoliation in the liquid phase, in which even after a long sonication treatment (few days) the concentration of dispersed graphene results very low (~ 0.01 mg/mL). The exfoliation process can not be employed in the large-scale production, so that, by now, the GO is obtained using graphite, which is subjected to thermal and ultrasound treatments in order to obtain GO-based thin films with the possibility to tune the electrical properties.

Today, the most commonly methods used to produce graphene oxide are: the Staudenmaier method, the Brodie method and the Hummers method^36–38^. The latter, that use concentrated sulfuric acids and highly oxidizing agents, has already become widespread to realize graphene oxide on large scale.

Moreover, the water-soluble GO allows further processing starting from the liquid phase, for example, spin coating and dip coating. The spin coating is also widely used to fabricate uniform thin films with controllable thickness^34,35^. To increase the absorption efficiency of the GO a thermal reduction treatment is used. On reduction, some of the GO oxygen groups are removed and bandgap can therefore be adjusted further by managing the oxygen present in rGO. Thus, reduced graphene oxide (rGO) behaves as semiconductor and its electrical conductivity may be tuned by controlling its oxygen content^39^.

In this work, a photodetector based rGO/n-Si heterojunction is presented to detect light radiation in a wavelength range from 375 to 920 nm. The rGO electrical properties ensure that, compared to commonly used transparent electrodes (eg. Indium Tin Oxide (ITO)) and ultrathin metals^12^, inside it the "hot photo-induced" charge carriers have a long life, especially in the UV wavelength range. These hot carriers contribute to the photocurrent of rGO which makes it easier to overcome the limits shown by traditional broadband silicon-based photodetectors.

In the realized rGO/Si based sensor the oxygen contained leak due to the air moisture, has been further reduced by using a cover made of poly-methyl methacrylate PMMA deposited on the top of the structure to insure its properties over time. In the experimental work presented here the feasibility of the rGO/n-Si device to detect UV light is demonstrated, so that it can be counted among the next generation of high-performance, cost-effective and ultra-low-power broadband photodetectors for innovative applications.

## Experimental details

**Figure 1:**
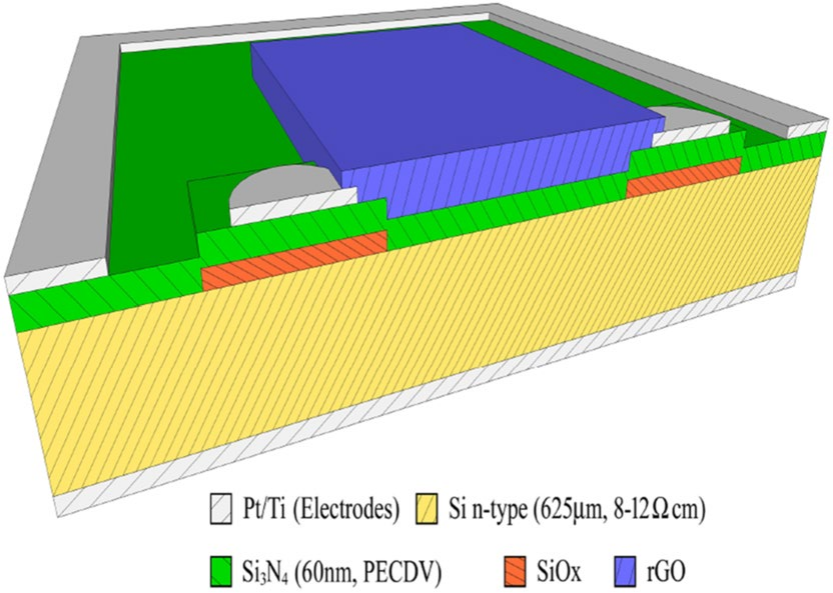
Schematic representation of the heterojunction cross section, formed by rGO (top surface) and Si (n-type) based substrate.

The graphene oxide (GO) in methanol was synthesized from graphite powder by a modified Hummers method. First, the GO solution (4% dispersed in distilled water produced by Graphenea Inc.^40^) was undergone to ultrasound treatment for about an hour to eliminate potential clusters. Then, a part of the solution was deposited on glass substrate by drop casting (GO layer), while the remaining solution was deposited on glass substrate by spin coating and thermally reduced (rGO layer). These two samples have been tested for a preliminary photoresponse analysis after that the fabrication process has been optimized to realize the photodetector reported in Fig. 1.

The photo-device was realized by deposition of reduced graphene oxide (rGO) layer on a silicon n-type substrate provided by FBK (Bruno Kesler Foundation)^41^. The rGO layer has been realized by spin coating and stripping procedure. First, the GO solution, after the ultrasound treatment, described above, was deposited on the sample in three steps each one consisting of a spin coating at 1500 rpm for 60 s and soft baking at 88 °C for 5 min. To reduce the GO film, the sample was heated on a controlled hot plate starting from 50 °C and increasing the temperature by 50 °C every 2 min up to 250 °C. After further 2 min, the hot plate is turned off removing the sample when the system reaches the room temperature. The rGO pad was defined by using an adhesive mask that was successively stripped together with the rGO film outside the desired area. In such a way, a rGO area down to 5 × 8 mm^2^ can be obtained, so that the photodetector active area is 40 mm^2^. Last, the device was mounted on the sample holder and wired then it was passivated using liquid PMMA (4%) by drop casting in order to preserve the rGO film from the weathering^42^.

Moreover, the substrates consist of an n-doped silicon wafer (resistivity of 8–12 Ω cm, 1 × 1 cm^2^ large, 625 μm thick) covered by a 60 nm thick insulating layer of silicon nitride (Si_3_N_4_) deposited by plasma-enhanced chemical vapour deposition (PECVD). Two circular Ti/Pt electrodes, 1 mm in diameter, are placed at a distance of 4 mm from each other on the silicon nitride surface. A 300 nm oxide layer under the electrodes improves their insulation. An integrated metallic ring, 1 mm large, closes off possible boundary currents. The bottom side of the silicon wafer is coated with Ti/Pt electrode. A 60 nm oxide layer under the electrode increases its insulation. More details on the substrate and the active layer used here are reported in the references^43–46^.

The UV–Vis–IR transmittance spectrum of the rGO thin film with and without PMMA coating has been carried out by using the Perkin-Elmar Lambda 2 spectrometer.

The photoresponse measurements have been carried out using a continuum wave (CW) laser diodes at wavelength of 378 nm, 405 nm, 550 nm, 685 nm, 785 nm, 805 nm, 920 nm with the laser power ranging from 0.1 to 1 mW. Furthermore, the I–V characteristics have been measured using a voltage supply (Source Meter Keithley, mod. 2635) and a picoammeter (Keithley Dual-channel, mod. 6482). In the heterojunction based device the voltage has been applied between the front and back electrodes, in reverse bias polarization, in order to reveal the current that drifts across the heterojunction formed by rGO\Insulator\n-Si.

## Results and discussion

**Figure 2:**
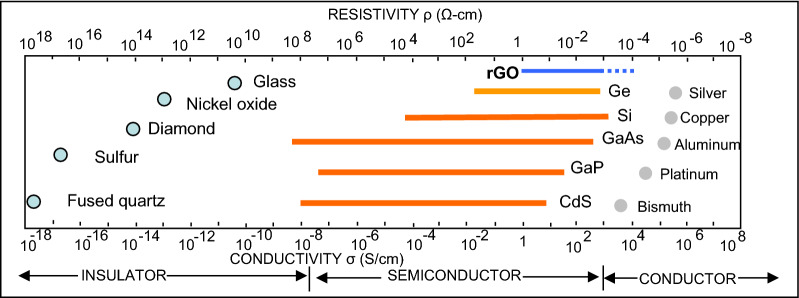
The rGO electrical properties compared with the main material used in the electro-optical applications.

Reduced graphene oxide shows interesting properties concerning the electrical conductivity. In Fig. 2 the comparison between the electrical properties of rGO and the principal material used in the optoelectronics, is reported.

It could be noted that GO, depending on its fabrication conditions, could be semiconductor or conductor with an electrical conductivity ranging between 1 S/cm down to 1 × 10^–4 ^S/cm. For this reason, a preliminary study of the GO thin film has been carried out, using a very low noise facility^47,48^. The GO-based samples used for the electrical investigation are represented by two narrow lines made of GO and rGO, respectively, with a length of 10 mm, a width of 1 mm and a thickness of 0.1 mm.

**Figure 3:**
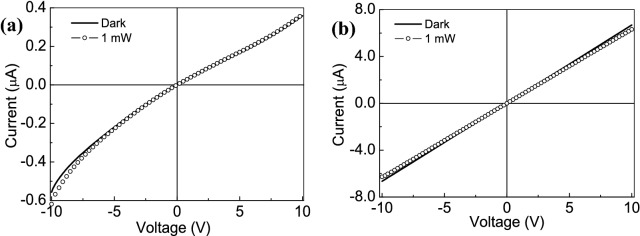
I–V characteristics of GO (**a**) and rGO (**b**), deposited on glass substrate and measured in dark conditions and using a 378 nm laser light tuned at 1mW.

In Fig. 3a the I–V curves of the GO layer deposited by drop-casting show a non-linear behavior addressed to semiconductor-like material with an electrical resistance of about 150 kΩ. There is a slight difference between the dark current and the current produced by illuminating with 1 mW laser light at 378 nm. In Fig. 3b the I–V characteristics measured on a line made of thermally reduced GO (rGO) show a linear behavior, it reproduces an ohmic-like characteristic having electrical resistance less than 300 Ω. A surface color change (not reported here) of the two GO-based samples has been observed: from the brownish-black of the GO to the brownish-yellow of the rGO thin films. This result depends on the rGO fabrication process as reported also by other authors^38^. In our experience, the film color is strictly dependent on the thermal reduction treatment used during the fabrication and on the electrical properties acquired by the reduced GO.

To better investigate the photoresponse capability of the GO and rGO thin films deposited on glass substrate, the current as a function of time was measured, by switching on and off the laser source at wavelength of 378 nm tuned at 1 mW.

**Figure 4:**
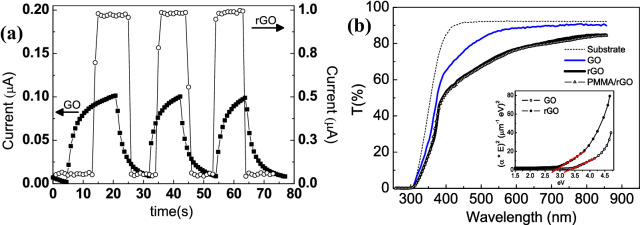
(**a**) Switching characteristics of GO and rGO samples irradiated with 378 nm at bias voltage of 5 V. In the graph three cycles in which the laser light is switched on and off are shown. (**b**) The percentage of optical transmittance of glass substrate, GO thin film, bare rGO and rGO covered by PMMA are compared. All the thin films are deposited on a glass substrate; (inset) Tauc’s plot analysis used to estimate the bandgap of GO and rGO. The red lines represent the fitting analysis used to obtain the band gap values.

In Fig. 4a the time dependent photocurrent at voltage of 5 V for the GO and rGO layer is reported. It could be noted that the rGO layer exhibits a higher current when the laser is switched on compared to the GO that shows a lower photoresponse as function of the laser light. Moreover, when the light is switched off the rGO recovers to the dark current value quicker (about tens of microseconds) than the GO (about 40 ms), suggesting that in the latter the conduction process is affected by trapping mechanism rather than the photogenerated carriers which prevails in the rGO sample. We speculate that in the GO sample the electron carriers are largely trapped by oxygenous groups under light radiation, resulting in a reduction of electron carrier density, whereas in the rGO sample the reduction process has decreased the presence of oxygenous groups, increasing the electron photogeneration^49,50^.

Moreover, the switching characteristic demonstrates that the rGO thin film is sensitive to the laser light, in particular it has photoresponse capability in the UV light spectral range.

Furthermore, one of the limitations that could affect the reduced graphene oxide is related to the oxygen contained leak when it is exposed to the environment i.e. the humidity absorption. This process could change the optoelectronic performance of the rGO thin film. For this reason, it is useful to cover the rGO thin film using a suitable coating material, to avoid the rGO change of optical and electrical properties. A possible solution that suits the purpose is represented by a poly-methyl methacrylate (PMMA) thin layer (few microns thick) deposited on the rGO thin film. The PMMA coating layer allows not only to protect the rGO from potential aging effects and oxygen leak, but it does not absorb UV incident light, so that it does not reduce the light intensity that the photodetector could absorb. In Fig. 4b the comparison between the optical transmittance of the glass substrate, the GO, rGO thin film with and without PMMA coating layer is reported.

It could be noted that the glass substrate has high transmittance starting from a wavelength of 350 nm so that it does not contribute to the optical response of the GO, rGO and PMMA/rGO layer, in the photoresponse measurements reported below. A more detailed look at Fig. 4b reveals that the transmittance curves of the PMMA/rGO and the rGO have the same trend, the presence of the PMMA layer affects the spectral transmittance only in the near infrared spectral range (where the difference between rGO and PMMA/rGO is about 1.5%), whereas in the UV range, the presence of PMMA coating layer is negligible (less than 0.5%). Moreover, the transmittance of GO, as reported in Fig. 4b, is higher than rGO over the spectral range 250–800 nm. This behavior entails that the band-gaps of GO and rGO are different. In our case, the Tauc’s plot analysis (reported in the inset of Fig. 4b) reveals for an allowed direct transition a bandgap of about 3.2 eV and 2.8 eV for GO and rGO thin film, respectively.

This result demonstrates that the reduction process used in this work has tuned the energy gap of the GO-based material, so that decreasing the GO oxidation degree it is possible to reduce the energy gap^50,51^.

Moreover, the low transmittance value of the PMMA/rGO thin film in the UV spectral range represents an important feature concerning the possibility to use these materials as light absorber in the optoelectronic applications. Let’s remark that rGO could be bear in mind as a suitable alternative to a photosensitive material like multi-walled carbon nanotubes (MW-CNTs), that has recently been proposed also for the detection of UV radiation^43,46^. Then, it is reasonable to consider the rGO thin film as an active material to realize broadband light sensors. With this aim a photodetector using thin film of reduced graphene-oxide/n-Si heterojunction, has been realized.

**Figure 5:**
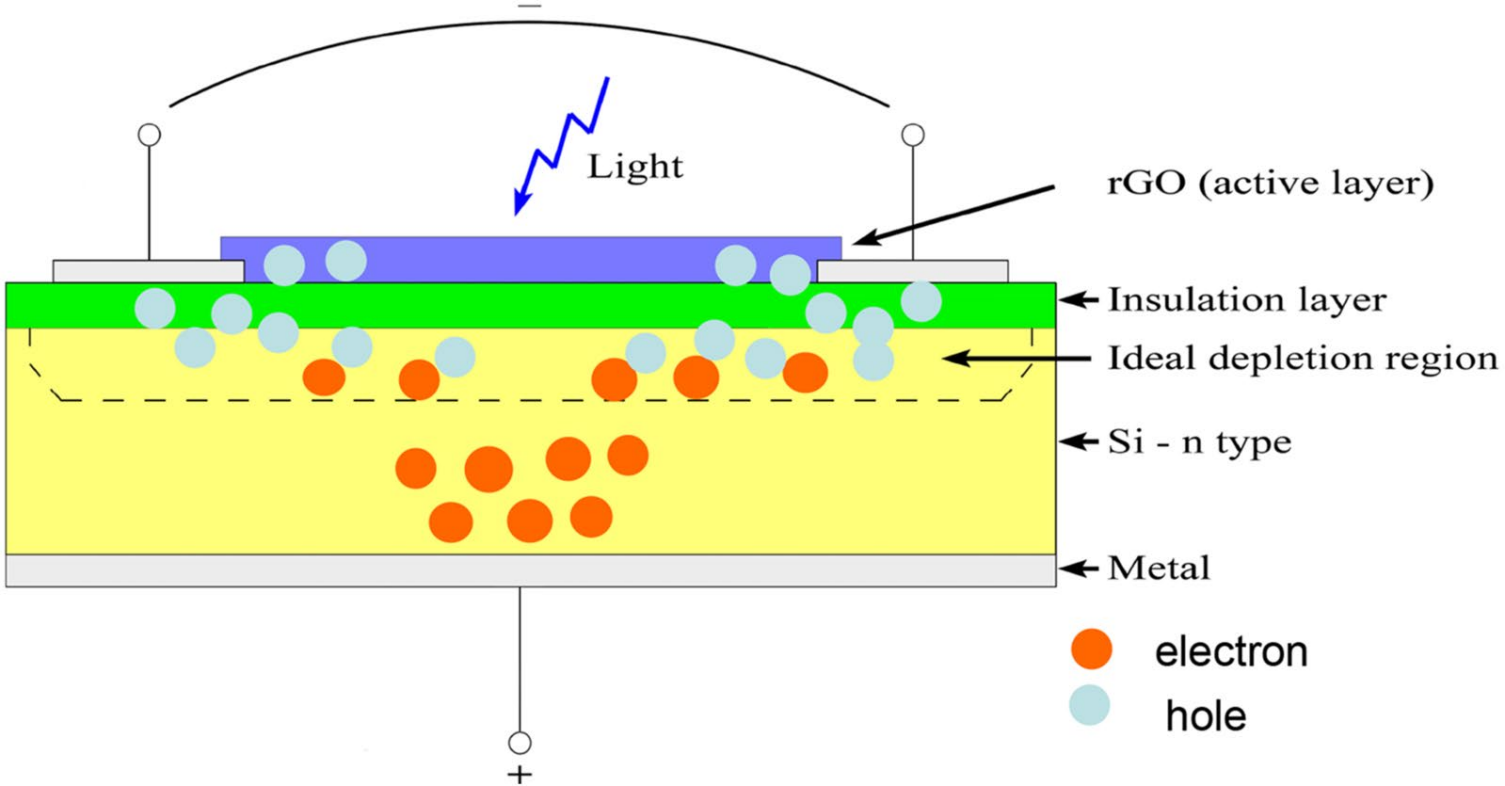
Schematic representation of the rGO/n-Si photodetector at reverse bias.

The sketch of the PMMA/rGO/n-Si heterojunction structure could be represented as reported in Fig. 5, where it is possible to point out from top to bottom the rGO layer, insulator layer, n-Si layer, the depletion area between rGO and Si layers, and the reverse bias voltage. Since the PMMA layer is not involved in any electrical process, it is not shown in Fig. 5 and in the following sections of the paper the PMMA/rGO/n-Si structure will be reported quite simply as rGO/n-Si.

Furthermore, as said before, rGO thin film shows semiconductor behavior so that it is reasonable to consider the structure rGO/n-Si as a heterojunction, in which the insulator is represented by a thin layer of Si_3_N_4_. If an external field is applied to heterojunction, the created carriers acquire a speed of drift: in particular the hole moves towards the negative electrode, the electrons towards the positive one. In the heterojunction there is a high electrical current only when it is in reverse polarization. This effect is due to the creation of the depletion region, that works as an active zone, in which the electron–hole pairs do not recombine but they are quickly set in motion, inducing the current in the electrodes and consequently in the external circuit. Therefore, the rGO/n-Si device has been polarized at reverse bias as shown in Fig. 5^52^.

**Figure 6:**
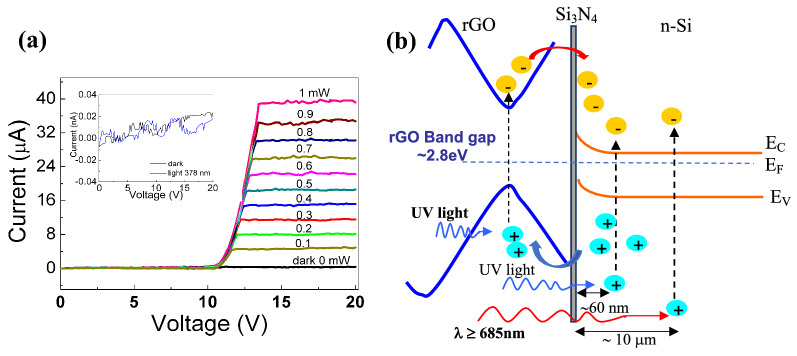
(**a**) I–V characteristics of the rGO/n-Si photodetector device. The photoconductive current has been measured in reverse bias configuration. (inset) the I–V characteristics of the n-Si based substrate with and without laser light at 378 nm; (**b**) a proposed energy band diagram of the rGO/n-Si heterojunction under light illumination at reverse bias. The rGO bandgap is about 2.8 eV. The UV (blue arrow) and infrared (red arrow) radiation are reported. The penetration depth in the n-Si is of the order of 60 nm for a wavelength of about 378 nm and 10 µm for a wavelength of 685 nm.

The current–voltage characteristics of rGO/n-Si heterojunction device illuminating with 378 nm laser diode light at different laser power is depicted in Fig. 6a. The measurements have been carried out inside a dark box using a diode laser with a spot diameter of 1 mm and with power ranging between 0.1 and 1 mW.

In Fig. 6a the current curves have the same trend: a very low value until about 11 V, where there is a threshold after that the current increases rapidly until it flattens out at value that depends on the laser power.

Moreover, the bare substrate under dark and light illuminating conditions (laser light at 378 nm) has been tested. The result is reported in the inset of Fig. 6a where the I–V characteristics demonstrate that in the n-Si based substrate the current is very low, about few tens of pico-ampere, and any photoresponse effect is present. Therefore, it could be asserted that the substrate does not contribute to the device photoresponse.

In addition, the current trend observed in Fig. 6a could be explained considering that increasing the reverse bias voltage the depletion region width increases lowering the electron–hole recombination at the interface. When the bias voltage exceeds the threshold value all the *photo-generated carriers* are collected at the electrodes so the depletion region is large enough to produce the highest current at the plateau.

Furthermore, the working principle of UV photodetector made by the heterojunction between reduced graphene oxide and silicon layer, can be understood using the relative band diagram, shown in Fig. 6b. When the rGO/n-Si photodetector is illuminated by light radiation, part of the light is absorbed by the rGO producing photo-excited electrons that cross the heterojunction interface, while the electron–hole pairs are created in the n-Si layer, so that holes across the interface move in the rGO. Finally, the carriers are collected from the electrodes contributing to the total photocurrent^53–55^.

In this work, the rGO layer of about 400 nm thick is deposited in a gently mode on the n-Si layer, thanks to the fabrication method used (a soft spin coating technique described in Sect. 2). In other words, the fabrication technique seems to reduce the formation of defects or damage in the region across the boundary of the two materials, as typically results using other deposition technique as PVD, sputtering etc. The presence of reduced graphene oxide on the top of the bulk semiconductor and the subsequent rearrangement of the interfacial energy band creates a light-sensitive junction having a thickness equal, at most, to just one atom below the surface (without impurities and growth imperfections). Uniquely, this architecture causes the rGO surface to be extremely close to the depletion region (for a conventional wafer-based pn junction it is typically buried several micrometers, i.e. below the surface), thus reducing the recombination of the hole-electrons pairs induced by the light radiation. On the other hand, silicon for incident light with wavelengths of 378 nm has large absorption coefficient but low penetration depth, about few hundreds of nanometers, so most of the photo-generated carriers are found near the silicon surface. Then, the ultrathin rGO/Si heterojunction is highly efficient in separating photo-generated carrier pairs, reducing the recombination of the carriers, which become photocurrent when the reverse bias voltage is higher than the threshold value.

These results demonstrate that the presence of rGO in the heterojunction suppresses the carriers recombination and produces further photo-generated carriers, resulting in the UV photoresponse of rGO/n-Si respect to the bare n-Si based substrate.

**Figure 7:**
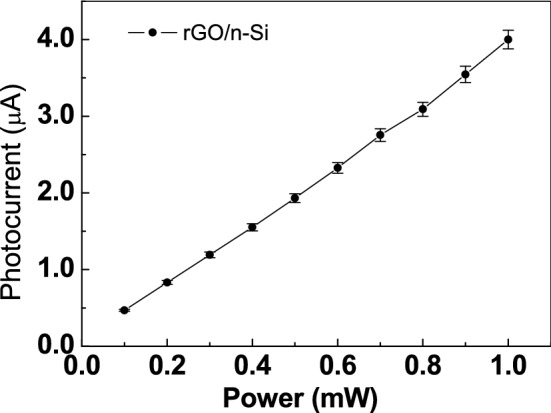
rGO/n-Si photocurrent at bias voltage of 25 V as a function of laser power.

Furthermore, the photocurrent at different laser power is analyzed as reported in Fig. 7.

The rGO photocurrent (the difference between the dark current and the one of illuminating device) is due to the illuminated laser power at 378 nm laser diode, tuned at power from 0.1 to 1 mW. Increasing the laser power the photocurrent increases linearly demonstrating that the rGO/n-Si based device is a suitable UV detector, offering some advantages respect to other material i.e. CNT^43^, such as cheaper and faster production process.

**Figure 8:**
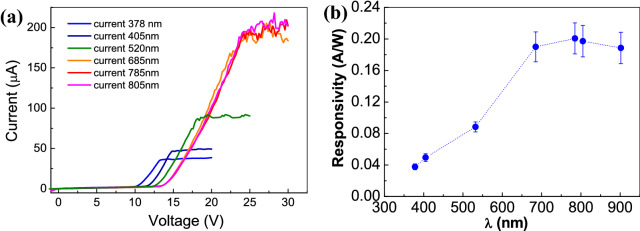
(**a**) I–V characteristics as a function of reverse bias voltage at laser light wavelengths from 378 to 805 nm; (**b**) rGO/n-Si responsivity versus light wavelengths.

The current characteristics of the rGO-based device as a function of different laser wavelengths, ranging from UV to IR spectral range, have been measured and reported in Fig. 8a. It could be noted that all the wavelengths produce the same current trend: at low voltage the curves have very low current, then beyond the voltage threshold, that depends on the laser wavelength, the current increases abruptly and finally it reaches the plateau. Clearly, the voltage threshold value increases as a function of laser wavelength, starting from 10 V at 378 nm and reaching about 14 V at 685 nm. It is interesting to note that starting from a wavelength of 685 nm there is an overlap through the I–V curves, a sort of saturation effect dependent on laser diode wavelength.

This effect could be addressed to the fact that the silicon penetration depth in the Vis-IR spectral range is about tens of microns. Then, in n-Si layer starting from 685 nm, the wavelengths generate hole-electron pairs also far away from the junction interface, in the depletion region, so that a very low recombination rate occurs. This effect produces an increasing of the depletion region width which is confirmed by the high voltage bias necessary to achieve the photocurrent plateau in the I–V characteristic at 685 nm^56^. Therefore, the generation of more electron–hole pairs together with low recombination rate produces more photo-generated carriers respect to the lower wavelengths, contributing to high value of the current plateau. Moreover, it could be noted that increasing the wavelength, the I–V characteristics at 685 nm, 785 nm and 805 nm are overlapped. It could be explained considering that the n-Si penetration depth at these wavelengths is only slightly increased, so that the number of photo-generated carriers is the same and a saturation condition is achieved. Moreover, at the plateau the current related to the 685 nm, 785 nm and 805 nm wavelengths shows some ripples that reveals a thermal effect probably because the current is mainly contributed by the thermally generation of electron–hole pairs in the n-Si layer depletion region.

In addition, the responsivity *R* = *I*_*ph*_/*P* (where *I*_*ph*_ is the photocurrent and P represents the laser power) of the realized rGO/n-Si device at 25 V of bias voltage has been evaluated. The responsivity measured at room temperature is reported in Fig. 8b. The rGO/n-Si based device shows a peak responsivity in the IR spectral range at around 750 nm with a corresponding value of 0.20 A/W in agreement with the data reported in literature^36^. The Quantum Efficiency (QE) has been carried out using the relation *QE* = *hcI*/*eλP* where *h* is the Plank constant, *e* the electron charge, *c* the light speed, *I*, *λ* and *P* represent the photocurrent at 25 V bias, the wavelength and the laser power, respectively. It must be noted that the QE (estimated at λ = 685 nm and P = 1 mW) is about 35%. Furthermore, the performance of several photodetector sensors reported in literature^26,36,43,49,57–59^ has been compared and collected in Table1. It could be noted that the rGO/n-Si based device reveals a responsivity value comparable with other graphene-based photodetectors, and a high value of photosensitivity (81.2 cm^2^/mW) and detectivity (7 × 10^10^ cmHz/W).

**Table 1:** Comparison on performances of photodetectors and rGO/n-Si based device. The responsivity of the device reported in the table refers to 685 nm light source.

Device structure	Responsivity (A/W)	Photosensitivity (cm^2^/mW)	Response range	Detectivity (cmHz/W)	I(Light)/I(Dark)	Ref
Bi2Te3/Si	1	10*E+3	370 nm–118.8 µm	4.7*E+10	–	^36^
GO junction	0.0236	0.024	290–1610 nm	3.31*E+7	–	^49^
G/Si	0.435	0.26	488–730 nm	1.4*E+8	–	^26^
GO/SiNW	0.009	3.4	532 nm–118.8 µm	–	–	^57^
Pd/G/TI	0.006	–	632–1550 nm	–	–	^58^
MWNT/n-Si heterojunction	0.1	1.89	370–815 nm	3.8*E+10	13.25*E+3	^43^
rGO/n-Si heterojunction	0.20	81.2	370–815 nm	7.02*E+10	3*E+5	This work
G-Bi2Te3	35	0.03	300–1600 nm	–	–	^59^

The results demonstrate that the rGO/n-Si based device is able to detect radiation from UV to IR wavelengths, in particular it enhances the UV radiations detection of conventional Si-based photodetector. Finally, further effort will be make with the aim of increasing the device responsivity in the UV spectral range, changing the energy profile of the rGO/n-Si, i.e. according with the technique used to design a quantum well profile.

## Conclusions

The photoresponsivity, in the UV–IR spectral range, of rGO/n-Si heterojunction fabricated by spin coating process have been reported. The electrical and optical characterization has demonstrated the semiconductive behavior of GO and rGO thin films with bandgaps of 3.2 eV and 2.8 eV, respectively. This result means that the reduction process reduces the bandgap improving the rGO photoresponse performance. In the UV spectral range the photocurrent shows a linear power dependence suggesting the capability of the realized rGO/n-Si heterojunction to detect also UV radiation.

Some figures-of-merit have been estimated and compared with other broadband photodetectors suggesting that the rGO/n-Si heterojunction has performances comparable with other devices. Under 685 nm illumination the rGO/n-Si heterojunction shows a quantum efficiency of 35%, while the responsivity, the photosensitivity and detectivity are 0.20 A/W, 81.2 cm^2^/mW, 7 × 10^10^ cmHz/W, respectively. These results demonstrate that the used fabrication process quickly and easily produces very uniform films, without damage the substrate and reducing the formation of defects/damage across the rGO/n-Si interface. In conclusion, the rGO/n-Si heterojunction reveals a broadband spectral response, opening interesting perspectives in the silicon device technology research field. Finally, the observed features, which are currently still under investigation, suggest the potential use of this device for optoelectronic applications.
